# Supplementary material for: Fabrication and Properties of the Multifunctional Rapid Wound Healing Panax notoginseng@Ag Electrospun Fiber Membrane
Source: Molecules. 2023 Mar 27;28(7):2972. doi: 10.3390/molecules28072972 (PMC10096071; doi:10.3390/molecules28072972)
Supplement: Supplementary file 1 [file molecules-28-02972-s001.zip › molecules-2251119-supplementary.docx]

**Supporting information**


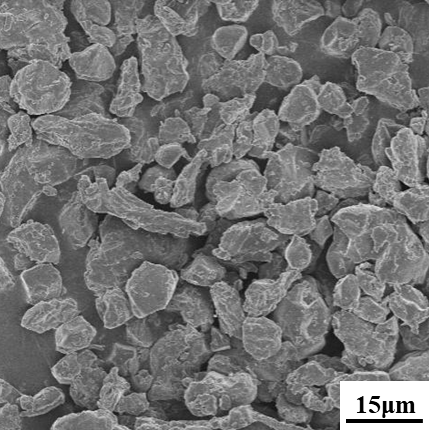


Fig. S1. The SEM image of the *Panax nototoginseng* (PN) powder.

From the SEM image of PN powder, it can be seen that the PN used for formulation of the electrospun fiber membrane has irregular shape. The size of PN particles is unevenly distributed, with the diameters between 2-15 μm.

####
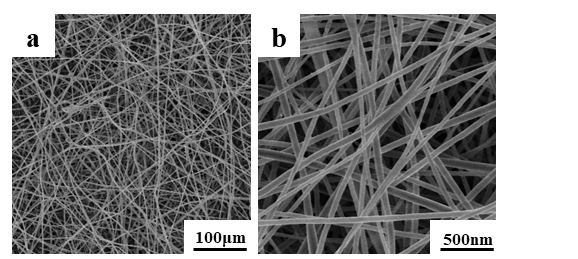


Fig. S2. SEM images of the PN@6 wt%Ag-2 h nanofiber membrane (a, b).

####
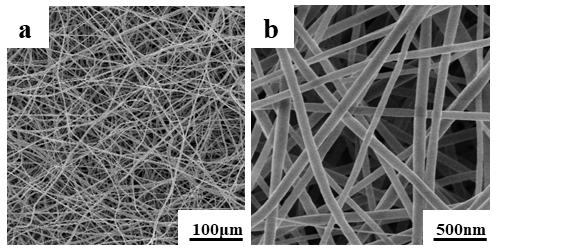


Fig. S3. SEM images of the PN@10 wt%Ag-2 h nanofiber membrane (a, b).


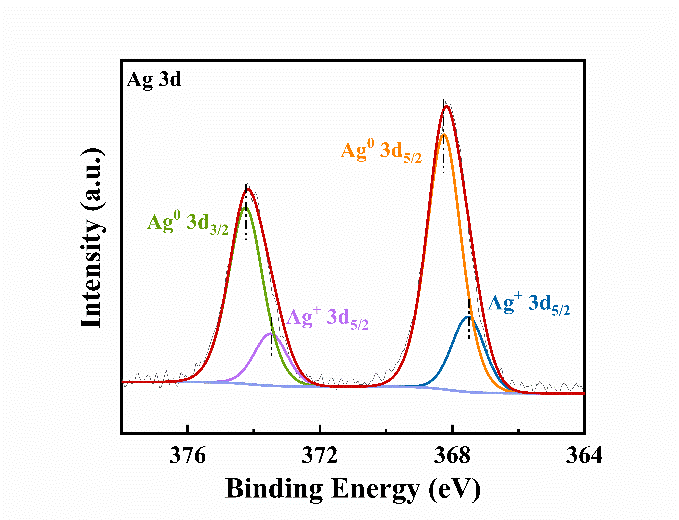


Fig. S4. XPS survey spectrum of PN@8 wt%Ag-2 h core/shell electrospun nanofiber membrane.

The X-ray photoelectron spectroscopy (XPS) of typical sample PN@8wt%Ag-2 h core/shell electrospun nanofiber membrane has been determined. The XPS spectra clearly show the elemental status of Ag 3d. The Ag 3d peaks are a doublet which arises from spin-orbit coupling (3d_5/2_ and 3d_3/2_). The peaks of Ag 3d level at 368.25 eV and 374.25 eV correspond to 3d_5/2_ and 3d_3/2_ states of Ag atoms respectively, and the peaks of Ag 3d level at 367.52 eV and 373.49 eV correspond to 3d_5/2_ and 3d_3/2_ states of Ag^+^ ions respectively. The results proved clearly that Ag exists in the form of Ag atoms and Ag^+^ ions, the amount of Ag atoms is 78.68 at%, the amount of Ag^+^ ions is 21.32 at%, indicating that the formation of Ag NPs in PN@8 wt%Ag-2 h core/shell electrospun nanofiber membranes.

Supporting information S5- *In vitro* drug release experiment

*In vitro* drug release experiment was carried out by the thermostatic shaking method. 0.20 g PN@8 wt%Ag-2 h electrospun fiber membrane and 15 mL PBS (pH=7.4) were put into the conical flask. The temperature of thermostatic shaker was 37 ℃, and the stirring speed was 135 rpm. At predetermined time (0.5, 1, 2, 4, 8, 12, 24, 36, 48 h), 3 mL release solution was successively taken from the conical flask, and an equal amount of PBS solution of the same pH and temperature was immediately supplemented.

The concentration of ginsenosides Rg1 in the release solution was determined by high performance liquid chromatography (HPLC). According to the standard curve of ginsenosides Rg1 in PBS, the cumulative drug release rate of Rg1 at different release time points was calculated. Each sample consisted of three parallel groups and the mean value was used for calculation. The calculation formula was shown in the following equation.

$$Q_{\text{n}}=V\times C_{\text{n}}+V_{1}\sum_{\text{i}=1}^{\text{n-}1} C_{\text{i}}\text{ n}\geq2$$

Where Q_n_ is the cumulative release amount of Rg1 (μg) at the Nth sampling, C_n_ and C_i_ are the concentration of Rg1 (μg/mL) in the released solution at the time of the Nth and the i sampling, V is the release amount (15 ml), and V_1_ is the volume of each sample (3 ml). According to the the percentage of Rg1 in PN, the cumulative drug release amount of PN at different release time points could be calculated.

After 48 h drug release, the PN@8 wt%Ag-2 h electrospun fiber membrane was removed from PBS and dried. Then the dried fiber membrane was dissolved in methanol. The amount of residual Rg1 in the membrane was calculated using HPLC with the standard curve of Rg1 in methanol. According to the the percentage of Rg1 in PN, the residual PN in the drug-loaded fiber membrane was calculated. Finally, the cumulative release rate of PN was calculated according to following equation.

$$\text{Cumualative release rate of PN}=\frac{\text{Cumualative release amount of }\text{PN}}{\text{Actual PN load amount}}\text{×100\%}$$

Where the actual PN load amount = the cumulative drug release amount (after 48 h) + the residual PN amount in the membrane.

The 48 h *in vitro* release curve of PN from the PN@8 wt%Ag-2 h electrospun nanofiber membrane was shown in Fig. S4. It can be seen that PN was gradually released from the PN@8 wt%Ag-2 h electrospun nanofiber membrane without burst release. The PN cumulative release rate of the PN@8 wt%Ag-2 h electrospun membrane was 65%.


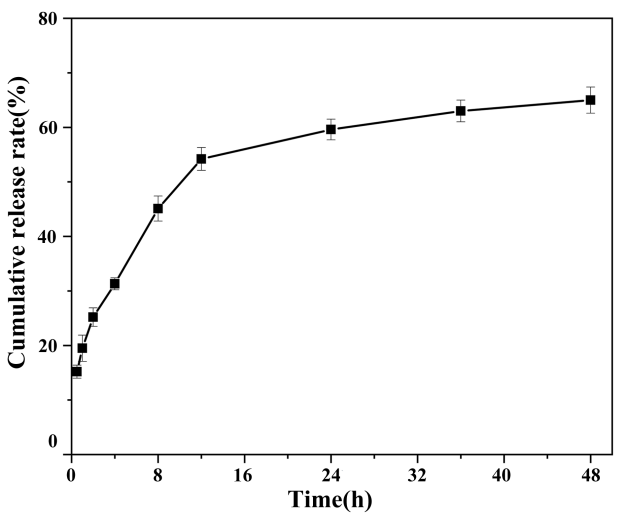


Fig. S5. *In vitro* PN cumulative release curves of the PN@8 wt%Ag-2 h core/shell electrospun nanofiber membrane.
